# Natural Regulatory T Cells in Malaria: Host or Parasite Allies?

**DOI:** 10.1371/journal.ppat.1000771

**Published:** 2010-04-29

**Authors:** Diana S. Hansen, Louis Schofield

**Affiliations:** The Walter and Eliza Hall Institute of Medical Research, Parkville, Victoria, Australia; University of California San Diego, United States of America

## Abstract

*Plasmodium falciparum* malaria causes 500 million clinical cases with approximately one million deaths each year. After many years of exposure, individuals living in endemic areas develop a form of clinical immunity to disease known as premunition, which is characterised by low parasite burdens rather than sterilising immunity. The reason why malaria parasites persist under a state of premunition is unknown but it has been suggested that suppression of protective immunity might be a mechanism leading to parasite persistence. Although acquired immunity limits the clinical impact of infection and provides protection against parasite replication, experimental evidence indicates that cell-mediated immune responses also result in detrimental inflammation and contribute to the aetiology of severe disease. Thus, an appropriate regulatory balance between protective immune responses and immune-mediated pathology is required for a favourable outcome of infection. As natural regulatory T (T_reg_) cells are identified as an immunosuppressive lineage able to modulate the magnitude of effector responses, several studies have investigated whether this cell population plays a role in balancing protective immunity and pathogenesis during malaria. The main findings to date are summarised in this review and the implication for the induction of pathogenesis and immunity to malaria is discussed.

## Natural Regulatory T Cells

Control of infection is the main function of the immune system. Some pathogens are difficult to eradicate and require a potent immune response involving destructive and cytotoxic mediators, which can result in adverse effects and immunopathology. Lymphoid populations exerting immunosuppressive activity, such as natural regulatory T (T_reg_) cells, have been shown to play a critical role in balancing protective immune responses and immune-mediated pathology [1]–[4]. This suppressor T cell linage is characterised by expression of high levels of CD25 and CD4 [5], while also expressing low levels of CD127 [6]. Natural T_reg_ cells constitutively express high levels of the T cell inhibitory receptor cytotoxic T lymphocyte–associated molecule 4 (CTLA-4) [7] and the glucocortcoid-inducible tumour necrosis factor receptor (GITR) [8]. Forkhead box P3 (Foxp3) is the only transcription factor required for generation of natural T_reg_ cells [9] and it constitutes the most specific marker for this cell population. Natural T_reg_ cells, initially identified by their capacity to inhibit the development of autoimmune gastritis [10], are now known to prevent other autoimmune conditions such as diabetes [7] and inflammatory bowel disease [2], [11], as well as inhibit anti-tumour immunity [12]. They have been shown to inhibit both IL-2 production and cell proliferation of conventional CD4^+^ T lymphocytes [13] by contact-dependent mechanisms and/or through the production of anti-inflammatory cytokines such as TGF-β [14] and IL-10 [15]. Although they have limited proliferative capacity and are unable to secrete IL-2, natural T_reg_ cells exert their suppressive function after stimulation through the T cell receptor, a process that appears to require antigen-presenting cells [13]. After activation, natural T_reg_ cells do no require further stimulation and are able to suppress T cell responses in a non-specific manner.

In addition to natural T_reg_ cells, induced T_reg_ cells constitute another subset of suppressor lymphocytes [16]. Unlike naturally occurring T_reg_ cells, which develop in the thymus, induced T_reg_ cells are derived from conventional CD4^+^ T cells and acquire suppressive activity upon activation and exposure to signals such as immunosuppressive cytokines or immature dendritic cells (DCs).

## Why Are T_reg_ Cells Expected to Play a Role in Malaria Infection?

Malaria is one the most serious infectious diseases of humans, infecting 5%–10% of the world's population, with approximately 500 million clinical cases annually. This infection is transmitted to vertebrate hosts by the bite of female *Anopheles* mosquitos that are infected with protozoan parasites of the genus *Plasmodium*. Although there are several different species of *Plasmodium* parasites, most cases of severe disease and fatalities are caused by the blood-stage cycle of *Plasmodium falciparum*, which is endemic in sub-Saharan Africa and throughout the tropics. The fatalities are associated with a spectrum of discrete and overlapping disease syndromes including acute respiratory distress, coagulopathy, shock, metabolic acidosis, hypoglycaemia, renal failure, pulmonary oedema, and cerebral involvement [17]. In endemic areas, the most susceptible population to symptoms associated with severe malaria are children under the age of 5 who have experienced few parasitic infections. After many years of exposure, individuals living in endemic areas develop clinical immunity to disease, which is characterised by low parasitemia levels rather than sterilising immunity [18], [19]. However, this clinical immunity is known as “premunition”, as it requires ongoing exposure to the pathogen, and is lost quite rapidly in the absence of transmission. The reason why malaria parasites persist under the state of premunition is unknown, but it has been suggested that immunosuppressive mechanisms able to modulate protective immunity might lead to the persistence of parasites. In support of this hypothesis, several immunosuppressive processes have been described accompanying both human and experimental malaria infections, including reduced T cell proliferative and IFN-γ responses to parasite antigens [20], [21] and production of immunosuppressive cytokines such as IL-10 [15], [22] and TGF-β [23]. As natural T_reg_ cells have been shown to mediate their suppressive effects through the production of these cytokines, it has been tempting to postulate that this immunoregulatory lineage could impede induction of immunity to malaria.

Although acquired host immunity limits the clinical impact of malaria infection and provides protection against parasite replication, experimental evidence indicates that cell-mediated immune responses also result in detrimental inflammation and contribute to induction of severe disease. In both *P. falciparum* infections and rodent malaria models, severe disease syndromes arise in diverse organs (e.g., brain, lungs, placenta) and appear to result from the combined effect of cytoadherence of parasitised red blood cells (pRBCs) in vascular beds and a strong host pro-inflammatory response mediated by cytokines such as TNF-α [24], LT-α [25], and IFN-γ [26], and effector cells such as CD4 [27], [28] and CD8 T cells [29], [30], natural killer (NK) T cells [31], [32], and NK cells [33]. As pro-inflammatory cytokines produced by these cells upregulate the expression of adhesion molecules such as intercellular cell adhesion molecule 1 (ICAM-1) [34], which is involved in the recognition of parasitic proteins expressed on pRBCs [35], it has been proposed that local and/or systemic inflammatory cascades exacerbate parasite sequestration. Furthermore, emerging evidence from both human malaria and experimental animal models [29], [30], [36], [37] has revealed the presence of leukocytes in blood vessels of inflamed tissue, suggesting that in addition to their systemic effects, intravascular infiltration of these cells might result in local inflammation and could also contribute to disease induction. Thus, cell-mediated immune responses appear to play a dual role in malaria: mediating protective immunity on the one hand, and contributing to severe inflammation and pathogenesis on the other. Whether natural T_reg_ cells are involved in promoting a balance between these processes has been the subject of several studies. In this review we have summarised the main findings to date investigating the role of natural T_reg_ cells during malaria, including experimental infection using a variety of murine infection models as well as human field studies. The implication of those findings for the induction of pathogenesis and immunity to malaria is discussed.

## Natural T_reg_ Cells and the Control of Parasite Clearance in Rodent Malaria Infection Models

Most of the studies using rodent models of infection have relied on the use of anti-CD25 antibodies for in vivo depletion of natural T_reg_ cells (Table 1). Although this appears to be a standard procedure to effectively deplete natural T_reg_ cells in vivo, one disadvantage of this approach is that CD25 is upregulated in activated T cells, thus making it difficult to specifically target natural T_reg_ cells in experimental models in which conventional lymphocytes are expected to become activated. This potential confounder should be addressed experimentally or taken into consideration for interpretation of experimental results.

**Table 1:** Effect of T_reg_ cell depletion in the outcome of malaria in rodent infection models.

Parasite	Mouse Strain	Effect on Parasitemia	Effect on Immune Responses	Effect on Severe Disease	Reference
*P. yoelii* 17X (PyL)	BALB/c	Reduction	Increased parasite-specific proliferation	Not determined	[38]
*P. yoelii* 17X (PyNL)	BALB/c	No effect	Not determined	Not determined	[38]
*P. yoelii* 17X (PyL)	C57BL/6	No effect	Not determined	No effect	[39]
*P. yoelii* 17X (PyL)	BALB/c	No effect	Not determined	No effect	[39]
*P. chabaudi adami* DS	BALB/c	Increase	Increased IFN-γ and TNF-α production	Increased haemolytic anaemia	[41]
*P. chabaudi adami* DK	BALB/c	No effect	Increased IFN-γ and TNF-α production	Increased haemolytic anaemia	[41]
*P. berghei* NK65	BALB/c	Delayed onset	Not determined	Not determined	[42]
*P. berghei*-ANKA	BALB/c	Reduction upon re-infection	Increased T_H_1 memory responses	Increased cerebral malaria rates upon re-infection	[53]
*P. berghei*-ANKA	C57BL/6	Not determined	Not determined	Protection from cerebral malaria when depletion was carried out 2 days before parasitic challenge. No effect when depletion was carried out 30 days before challenge.	[55]
*P. berghei*-ANKA	C57BL/6	Reduction	Enhanced T cell activation and IFN-γ responses	Protection from cerebral malaria when depletion was carried out 2 and 14 days prior to parasitic challenge	[54]
*P. berghei*-ANKA	CBA	Reduction	Not determined	Protection from cerebral malaria when depletion was carried out 14 days prior to challenge	[56]
*P. berghei*-ANKA	C57BL/6	No effect	Enhanced T cell activation	No effect	[56]

Several studies have investigated whether natural T_reg_ cells modulate the induction of immune mechanisms responsible for the control of parasite burden. Experimental infections with malaria species such as *Plasmodium chabaudi* and *Plasmodium yoelii* are particularly useful for addressing this question since they result in high parasitemia levels and do not induce T cell–mediated organ-specific disease syndromes (Table 2). There are two sub-strains of *P. yoelii* 17X. One of them is highly virulent (PyL) and induces lethal infections, whereas the other one (PyNL) results in a self-resolving non-lethal infection. This difference in outcome suggested that while infection with non-lethal parasites induces adaptive immune responses able to limit parasite replication, immunosuppressive mechanisms might operate when mice are infected with the lethal sub-strain, preventing the induction of protective immunity. Two independent studies have investigated whether natural T_reg_ cells play a role in the inhibition of immune responses against *P. yoelii* 17X. One found that depletion of natural T_reg_ cells protects BALB/c mice from overwhelming parasitemia and death after infection with PyL parasites, raising the possibility that natural T_reg_ cell function provides an essential escape mechanism used by malaria parasites to evade host-mediated immunity [38]. A subsequent study found that Foxp3^+^ regulatory T cells expand and become activated in response to infection with both PyL and PyNL parasites. However, in vivo depletion experiments failed to recapitulate the initial observations supporting a role for natural T_reg_ cells in the control of parasitemia levels [39]. Moreover, CD4^+^ CD25^−^ Foxp3^−^CD127^−^ adaptive T cells and not natural T_reg_ cells were found to be the source of IL-10 responsible for downregulation of protective immune responses during *P. yoelii* lethal infections [39]. The reasons for this inconsistency are unclear. The composition of intestinal flora has been shown to influence the development of natural T_reg_ cells [40]. It is therefore possible that T_reg_ cell depletion in genetically identical mouse strains housed in various animal facilities could lead to different infection outcomes if such mice differ in the composition of bacteria in their gastrointestinal tract.

**Table 2:** Malaria rodent models used to investigate T_reg_ cell function.

	Lethal Infection		Severe Immunopathology	
	BALB/c	C57BL/6	BALB/c	C57BL/6
*P. yoelii* 17X (PyL)	Yes	Yes	No	No
*P. yoelii* 17X (PyNL)	No	No	No	No
*P. chabaudi adami* (DS)	Yes	No	No	No
*P. chabaudi adami* (DK)	No	No	No	No
*P. berghei* NK65	Yes	Yes	No	No
*P. berghei*-ANKA	Yes	Yes	No	Yes

**Figure 1:**
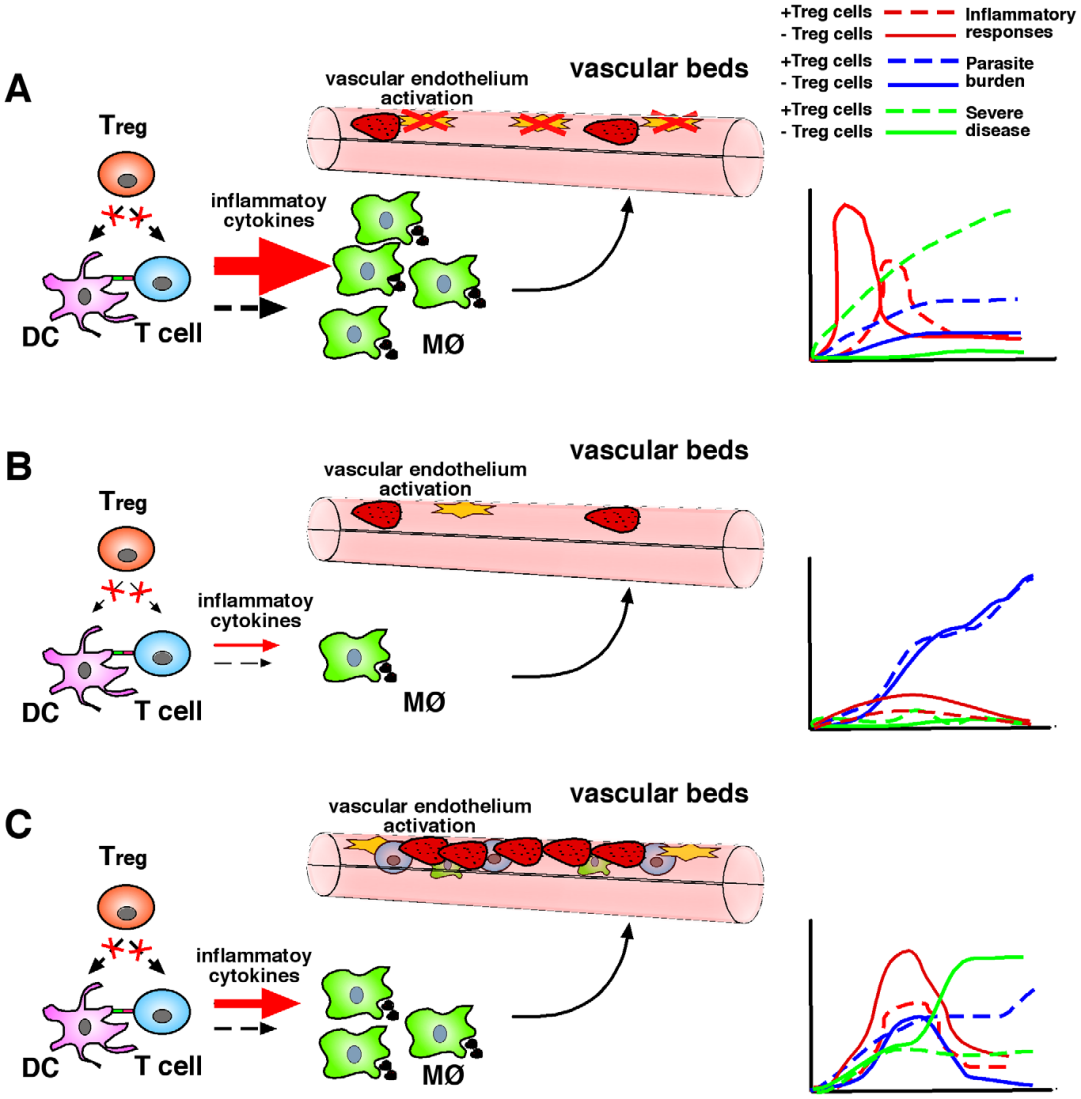
T_reg_ cells in malaria: Lessons learnt from experimental rodent models. (**A**) In murine models characterised by high pro-inflammatory responses to infection, T_reg_ cell depletion may result in a strong and rapid cellular immune response, which could rapidly control parasitemia, in turn reducing parasite-mediated pathology. MØ, macrophage. (**B**) In an infection setting resulting in poor inflammatory responses to malaria, a lack of T_reg_ cells may improve the induction of such responses but at levels that are not sufficient to control parasite burden or induce immunopathology. (**C**) In re-infection models, T_H_1 responses generated in the absence of T_reg_ cells during primary exposure appear to be robust enough to facilitate control of parasitemia in a secondary challenge at the expense of increased immune-mediated severe disease induction.

The impact of natural T_reg_ cell depletion was also evaluated in infection of mice with lethal (DS) and non-lethal (DK) strains of *P. chabaudi adami*. T_reg_ cell depletion resulted in increased production of IFN-γ and TNF-α in response to both lethal and non-lethal *P. chabaudi adami* infection. In spite of the enhancement of pro-inflammatory responses, T_reg_ cell depletion did not alter the course of infection in animals challenged with non-lethal parasites [41]. Curiously, T_reg_ cell depletion was found to exacerbate parasite burden and accelerate death in mice infected with the lethal DS strain. The haemolytic anaemia that results from hyperparasitemia was also enhanced in the absence of natural T_reg_ cells. These studies accord with the notion that in experimental models resulting in parasitemia of high density, T helper cell 1 (T_H_1) responses are not sufficient to control parasite burden, and consistently the T_reg_ cell–dependent modulation of pro-inflammatory responses is not critical in determining infection outcome (Figure 1B).

The effect of natural T_reg_ cell depletion with anti-CD25 antibody was also evaluated in immunised as well as naïve BALB/c mice after infection with *P. berghei* NK65. T_reg_ cell depletion resulted in a delay in the onset of parasitemia compared to non-depleted controls [42]. Whether T_reg_ cell depletion influenced the development of immune responses involved in the control of parasitemia was not investigated.

## The Role of Natural T_reg_ Cells in the Control of Experimental Cerebral Malaria

Although infection of mice with *P. yoelii*, *P. berghei* NK65, and *P. chabaudi adami* results in hyperparasitemia and death of susceptible mice due to haemolytic anaemia, it does not induce T cell–mediated organ-specific immunopathology. Therefore, a contribution of T_reg_ cells to the control of pathogenic immune responses cannot be accurately assessed in those infection models. In contrast, infection of susceptible mouse strains with *Plasmodium berghei*-ANKA induces cerebral malaria (Table 2). This murine infection has many features in common with the human disease and is thus an accepted model for certain important aspects of clinical malaria [43]–[46]. It manifests an inflammatory cytokine–dependent encephalopathy associated with upregulation of adhesion molecules on the cerebral microvascular endothelium, which facilitates sequestration of pRBCs and the resulting recruitment of inflammatory CXCR3^+^ leukocytes [33], [47], [48]. C57BL/6 mice, genetically predisposed towards T_H_1-dominated responses [49], are susceptible to the murine cerebral malaria syndrome, whereas BALB/c mice, with a genetically determined bias towards T_H_2 responses, are resistant [50]. Moreover, C57BL/6 and BALB/c mouse strains differ in the expression of molecules encoded by a polymorphic genetic region called the natural killer complex (NKC) [51], and it has been shown that the differential expression of these receptors in NK cells and CD1d-restricted NKT cells influences their immunological behaviour in response to malaria infection and accounts for the degree of susceptibility to severe malaria [31], [32]. C57BL/6 NKC alleles are associated with disease susceptibility and the increased severity to cerebral malaria is associated with a differential immune response to infection, characterised by a significantly enhanced IFN-γ production [32], [52]. Interestingly, anti-inflammatory cytokines such as TGF-β and IL-10 have been found to regulate type-1 responses during infection [23] and to play a protective role against *P. berghei*–mediated cerebral malaria [22], respectively, suggesting that resistance to disease could be associated with the development of suppressive mechanisms aimed at directly inhibiting pathogenic inflammatory responses. Specifically to test that proposition, we investigated the induction of T cell responses in cerebral malaria–resistant mouse strains. Our main results revealed that CD4^+^ T cells from cerebral disease–resistant BALB/c mice display a strong inhibition of cell proliferation and IL-2 secretion in response to both parasite-specific and polyclonal stimuli and that natural T_reg_ cells appear to play a role in the inhibition of T cell function occurring during infection [53]. Moreover, anti-CD25 depletion experiments revealed that T_reg_ cells also prevent the development of parasite-specific T_H_1 memory cells involved in the induction of cerebral malaria during a secondary parasitic challenge, demonstrating a regulatory role for this cell population in the control of pathogenic responses leading to fatal disease [53]. Interestingly, although the lack of natural T_reg_ cells during primary exposure to malaria enhanced T_H_1 memory responses and increased disease severity during re-infection, it also resulted in improved control of parasite burden. Thus, the results support the notion that although T_H_1 responses could be beneficial to control malaria re-infection, they should be carefully regulated in order to prevent harmful pathogenesis (Figure 1C).

Two independent studies investigated the effect of natural T_reg_ cell depletion with anti-CD25 antibody in the development of cerebral malaria using the disease-susceptible C57BL/6 mouse strain. Because both CD4 and CD8 T cells have been shown to contribute to the induction of severe malaria in this mouse strain, the use of anti-CD25 antibodies in T_reg_ cell depletion protocols requires careful experimental design to avoid targeting activated T cells, which could confound interpretation of the results. Both studies evaluated susceptibility rates of C57BL/6 mice that had been depleted of natural T_reg_ a day prior to infection with *P. berghei*-ANKA [54], [55]. Under these experimental conditions, anti-CD25 injection resulted in complete protection from cerebral malaria. To minimize depletion of parasite-specific activated T cells that could be potentially involved in cerebral malaria induction, both studies performed T_reg_ cell depletion several weeks prior to parasitic challenge. Whereas T_reg_ cell depletion 30 days before challenge did not alter parasite burden and susceptibility rates of C57BL/6 mice to cerebral malaria [55], anti-CD25 treatment carried out 14 days before parasitic challenge appeared to protect susceptible mice from fatality [54]. Similar results were obtained when natural T_reg_ cells were depleted under similar conditions in cerebral malaria–susceptible CBA mice [56]. Protection from disease was associated with reduced parasite burden and reduced sequestration of pRBCs in brains of infected mice [54]. The increased resistance to infection correlated with enhanced levels of T cell activation and IFN-γ production in spleens of *P. berghei*-ANKA-infected mice. These latter data suggest that administration of anti-CD25 antibody does not lead to significant depletion of activated antigen-specific T cells in these experiments. However, a recent study shows that T_reg_ cell depletion in DEREG mice, which are transgenic for a bacterial chromosome expressing a diphtheria toxin (DT) receptor–enhanced GFP fusion protein under the control of the *foxp3* locus that allows for selective depletion of Foxp3^+^ T_reg_ cells by DT injection, did not alter the incidence of cerebral malaria after acute *P. bergei*-ANKA infection [57].

Thus, the contribution of T_reg_ cells to the induction of cerebral malaria in the C57BL/6 background remains controversial. If a role in acute responses is proven, it might be the case that strong early IFN-γ responses produced in the absence of T_reg_ cells in disease-susceptible C57BL/6 mice facilitate control of parasite burden, resulting in reduced sequestration and protection from cerebral malaria (Figure 1A). The magnitude of inflammatory responses obtained after T_reg_ cell depletion in disease-resistant BALB/c animals (genetically predisposed to produce poor IFN-γ responses to malaria [52]) results in a more modest effect, which only exacerbates disease severity but is not sufficient to control parasitemia or increase fatality rates in a primary infection. In cerebral malaria–resistant animals, the impact of T_reg_ cell depletion becomes evident after re-infection, as T_H_1 memory responses generated in the absence of T_reg_ cells during initial exposure appear to be robust enough to control re-infection despite increased cerebral malaria induction rates. These considerations imply that natural T_reg_ cell function could be either beneficial or detrimental depending on the timing and magnitude of pro-inflammatory responses to malaria and the genetic background of the host.

## Human Studies

A few studies have investigated the role of natural T_reg_ cells in human malaria (Table 3). The implementation of such human studies is quite complex. A variety of different factors, including study design, number of individuals recruited for the study cohort, and use of fresh or cryopreserved samples may all have an impact on the results. These variables should be taken into account when assessing the relevance of conclusions arising from human disease association studies.

**Table 3:** Association between natural T_reg_ cells and the outcome of human malaria infections.

Population	Expansion in Infection	Association	Immunosuppressive Activity	Reference
Experimental sporozoite infection of volunteers	Frequency of T_reg_ cells increased after experimental exposure	Percentage of T_reg_ cells in PBMCs inversely correlated with onset of high parasite density	T_reg_ cells from infected volunteers were found to inhibit in vitro proliferative responses	[58]
Volunteers from Kenya	Not determined	Increased T_reg_ cell numbers in peripheral blood was associated with increased risk of clinical malaria	Not determined	[59]
Fulani and Mossi volunteers	Percentage of T_reg_ cells was higher in Mossi than Fulani individuals	High resistance to malaria in Fulani individuals correlates with a functional deficit of T_reg_ cells	Lower immunosuppressive activity in malaria-resistant Fulani individuals	[60]
Gambian children with severe or uncomplicated malaria	Increased number of T_reg_ cells in severe and uncomplicated malaria during convalescence	Frequency of T_reg_ cells negatively associated with the magnitude of T_H_1 memory responses	Similar immunosuppressive function in cases with severe and uncomplicated malaria	[61]
Adult volunteers from Papua with severe or uncomplicated malaria	Increased number of T_reg_ cell in malaria patients relative to asymptomatic controls	T_reg_ cell frequency associated with parasite biomass in severe but not uncomplicated malaria cases	Severe malaria cases were characterised by occurrence of a T_reg_ cell subset expressing high levels of TNFRII and Foxp3 with strong immunosuppressive function	[62]
Volunteers with acute *P. vivax* infection in Thailand	Significant expansion during acute infection	Not determined	Not determined	[65]

Foxp3 mRNA expression levels as well as CD3^+^CD4^+^CD25^hi^CD69^−^ cell numbers were found to become highly upregulated after experimental sporozoite infection of volunteers via mosquito bites. Interestingly, individuals with a high percentage of CD4^+^ T_reg_ cells amongst their peripheral blood mononuclear cells (PBMCs) were found to reach high parasite densities earlier than those with low T_reg_ cell frequencies, suggesting that the presence of T_reg_ cells in peripheral blood from infected individuals might facilitate blood-stage parasite replication. Reduced production of the pro-inflammatory cytokines IFN-γ, IL6, and IL-12 as well as significantly enhanced production of TGF-β levels were also found to be associated with high parasite burden [58]. Interestingly, monocytes and not T_reg_ cells were found to be the cellular source of TGF-β produced in response to infection. The authors proposed that monocyte-derived TGF-β might favour T_reg_ cell differentiation by inducing Foxp3 expression in naïve peripheral CD4^+^ T lymphocytes. Increased numbers of natural T_reg_ cells would in turn downregulate pro-inflammatory responses, which could result in enhanced parasite growth [58].

The frequency of natural T_reg_ cells was also investigated in naturally infected individuals in a malaria-endemic region of coastal Kenya. Multivariate analysis showed a significant association between increased CD4^+^ CD25^high^ numbers in peripheral blood and increased risk of clinical malaria [59]. The majority of CD4^+^ CD25^high^ cells were found to express Foxp3, suggesting that natural T_reg_ cells may negatively affect natural acquired immunity to malaria.

Interethnic comparative approaches on the susceptibility to malaria performed in West Africa revealed that despite similar levels of exposure, Fulani individuals appear to be more resistant to *P. falciparum* malaria, as they display reduced parasitemia levels and lower incidence of clinical episodes than other ethnic groups. When the expression of genes involved in immune responses was analysed in PBMCs of Fulani and sympatric Mossi individuals, increased expression of T_H_1 (IFN-γ, IL-18, TBX21) as well as T_H_2-related genes (IL-4, IL-9, GATA3) was detected in the more resistant Fulani individuals [60]. Interestingly, the upregulation of immunity-associated genes correlated with reduced frequencies of CD4^+^ CD25^+^ Foxp3^+^ cells in peripheral blood in Fulani compared to Mossi individuals and reduced expression of T_reg_ cell–related products such as CTLA4 and TGF-β. Moreover, whereas in vitro depletion of CD25^+^ cells enhanced proliferative responses to *P. falciparum* antigens in PBMCs from Mossi individuals, T_reg_ cell depletion did not alter parasite-specific proliferation rates of the Fulani, suggesting that a functional deficit of natural T_reg_ cells could be involved in the lower susceptibility to malaria of this ethnic group [60].

The role of natural T_reg_ cells in clinical malaria was investigated in children with either severe or uncomplicated malaria [61]. T_H_1 responses were found to be more elevated in severe compared to uncomplicated malaria cases. However, no significant differences in the absolute number or the frequency of natural T_reg_ cells were detected between these two groups. Moreover, in vitro depletion studies with anti-CD25 resulted in similarly increased cell proliferation to schizont antigen in both severe and uncomplicated malaria cases, suggesting that T_reg_ cells from both groups of patients have similar activity and implying that natural T_reg_ cell function is not sufficient to downregulate the strong T_H_1 response to infection occurring in children with severe malaria. Interestingly, high *FOXP3* mRNA levels at base line were found to be associated with reduced parasite-specific IFN-γ memory responses amongst PBMCs collected form convalescent malaria patients [61]. This result resembles findings obtained in the *P. berghei*-ANKA mouse model of severe malaria [47]. Whether T_reg_ cells acquired during a primary infection in human malaria are able to modulate T_H_1 memory responses involved in parasite clearance without inducing immunopathology requires further investigation.

The relationship between T_reg_ cell function and parasite biomass was evaluated in adults with uncomplicated or severe malaria in an endemic area of West Papua [62]. T_reg_ cell numbers increased in malaria cases compared to non-infected controls, indicating that these cells expand in response to infection. In patients with severe but not uncomplicated malaria, T_reg_ cell frequency was correlated with parasite biomass. Phenotypic characterization revealed that T_reg_ cells from severe malaria cases express high levels of TNFRII and constitute a subset of regulatory cells that express high levels of Foxp3 and appear to exert strong immunosuppressive function [62].

Recent in vitro studies using PBMCs from healthy donors stimulated with *P. falciparum*–infected RBCs have provided interesting insights into the mechanism by which T_reg_ cells are induced by malaria [63]. Stimulation with parasitised RBCs was found to induce expansion of two T_reg_ cell populations expressing different levels of Foxp3. Only cells expressing intermediate (Foxp3^int^) but not high (Foxp3^hi^) Foxp3 levels were found to secrete large amounts of effector cytokines. Moreover, high proportions of Foxp3^hi^ cells in the co-culture systems correlated with reduced production of pro-inflammatory cytokines, which suggests that this subset plays a role in inhibition of parasite-induced effector responses. Both malaria-elicited T_reg_ subsets appeared to be derived from conventional CD4^+^CD25^−^Foxp3^−^ T cells. The parasite-driven upregulation of Foxp3 levels required activated monocytes. However, soluble factors rather than direct contact were found to be responsible for this process. These results suggest a T cell receptor–independent mechanism for bystander activation of CD4^+^CD25^+^Foxp3^+^ T cells during malaria.

Phenotypic characterization of T_reg_ cells in PBMCs of healthy Gambian donors ex vivo revealed a very heterogenous population of Foxp3^+^ cells in this cohort study, with cells displaying different expression levels of CD25 [64]. In vitro stimulation with crude parasite extracts increased CD25 expression levels. The memory phenotype of CD4^+^Foxp3^+^ T cells was also investigated using expression of the chemokine receptors CCR7 and CCR4 to identify central and effector memory cells, respectively [64]. Ex vivo analysis indicated that antigen-experienced (CD45RO^+^) Foxp3^+^ cells were predominantly effector memory cells. Moreover, these cells were also found to express high levels of CD95 consistent with a highly pro-apoptotic phenotype.

Only one study has investigated whether T_reg_ cells become activated in response to *Plasmodium vivax* infection. Similar to association studies in *P. falciparum* malaria, the percentage of Foxp3^+^ T_reg_ cells was found to increase in acute *P. vivax* infection. In vitro elicitation assays confirmed this observation, as the number of Foxp3^+^ cells amongst PBMCs significantly increases in response to *P. vivax* antigens [65]. The relevance of these findings in the context of *P. vivax* malaria needs to be further investigated.

## Concluding Remarks

The current evidence from rodent infection models as well as human studies suggests that the natural T_reg_ cell pool becomes activated and expands in response to malaria infection [39], [41], [55], [61], [62], [66]. Whether such expansion results from direct proliferation, cytokine-driven differentiation, and/or recruitment of natural T_reg_ cells to secondary lymphoid organs is not completely understood. In vitro studies provided evidence that T_reg_ cells proliferate in response to malarial antigens [62], [65]. The precise mechanism by which such antigens stimulate natural T_reg_ cells is still unclear. It has been shown that T_reg_ cells express toll-like receptors (TLRs) and proliferate in response to lipopolysaccharide [67], raising the possibility that putative malarial TLR agonists such as GPI [68], [69], [70] or hemozoin-bound DNA [71] might stimulate T_reg_ function during infection. Although it is not known if these molecules can specifically stimulate proliferation upon binding to natural T_reg_ cell–expressing TLRs, a recent study demonstrated that TLR9 engagement in DCs is required for natural T_reg_ cell activation by malaria parasites [72].

Although the available literature does not support a unified model for the role of natural T_reg_ cells in malaria, a common feature emerging from data in experimental models and human association studies appears to be the ability of this suppressive T cell population to modulate acute pro-inflammatory and/or T_H_1 memory responses to infection. In animal models, the increased inflammatory response resulting from natural T_reg_ cell depletion was not always sufficient to control parasitemia levels, particularly in models of high parasite density in which antibody-mediated responses are known to play an important role in protective immunity. In low parasite density models, though, pro-inflammatory responses generated in the absence of natural T_reg_ cells appeared to be more efficient in controlling acute infection, leading to reduced organ-specific parasite sequestration. Consistent with an immunoregulatory role in chronic conditions [73], [74], evidence arising from rodent re-infection models [53] as well as severe malaria association studies in humans [61] supports the notion that natural T_reg_ cells modulate parasite-specific T_H_1 memory rather than acute responses involved in disease induction, suggesting that this cell population may limit the risk of immune-mediated pathogenesis upon re-infection. On the other hand, human studies available to date reveal associations between T_reg_ cell frequency and increased parasite densities, which in some cases were found to correlate with increased severity of disease. Overall, the literature suggests that whereas in some settings T_reg_ cells might modulate detrimental inflammation involved in disease induction, in other cases T_reg_ cell–mediated inhibition of immune responses results in increased parasite density, which is also known to be a key determinant of disease severity. As often in malaria, the picture seems to be more complex than initially envisaged, and whether natural T_reg_ cell activation results in a favourable outcome for the parasite or the host depends on a variety of factors, including the timing and magnitude of such pro-inflammatory responses to infection, the level of exposure to the parasite, and the genetic background of the host.

The findings concerning the role of T_reg_ cells in relation to malaria infection thus appear consistent with the growing literature on the role of T_reg_ cells in other infectious diseases, supporting the general view that these cells maintain homeostasis by controlling the magnitude of immune responses. Broadly speaking, in the context of other infectious diseases, T_reg_ cells curtail potent and sometimes over-vigorous effector responses. The selective basis of this activity appears to be the need to limit the significant tissue damage that can arise as a side-consequence of antimicrobial immune responses. For example, reactive oxygen species, while a powerful weapon against microbes, can cause significant damage to the host. In certain circumstances, the need to reduce or prevent serious tissue damage can compromise the ability to control infection. Examples of the role of T_reg_ in reducing immunopathology can be found in the original observation of their role in control of colitis [75], reduction of pulmonary inflammation in *Pneumocystis* [76], control of hepatic pathology in *Schistosoma* infections [77] and control of immunopathological leasions in viral infections [78], etc. The evidence for T_reg_ cell–dependent control of immune responses, leading sometimes to failure of the effector arm, comes most clearly from studies on chronic infections e.g., *Leishmania major* [79]. One outcome of the counter-regulatory activity against the effector response appears to be the promotion of pathogen persistence, as exemplified by *Litomosoides* [80]. T_reg_ cells may thus play a crucial role in maintaining long-term, balanced host/pathogen relationships, and hence in the promotion of commensalism.

## Future Directions

Future directions in T_reg_ cell research of relevance to malaria can be envisaged at several levels. Firstly, the activation of T_reg_ cell or their induction in the periphery appears to result from a wide range of signals, including direct stimulation of TLR or other pattern-recognition receptors, the maturation and activation of DCs and other accessory cells by pathogens through similar pathways, the cellular cytokine milieu and the potential contribution of parasite ligands with direct regulatory roles. For mechanistic studies, much can be attempted with human and murine cells in vitro, and in murine models in vivo, to analyse the molecular signals of parasite origin leading to T_reg_ cell activation, and the other contributory cells of the immune system. Secondly, as exemplified here, the contribution of T_reg_ cells depends upon the kinetics and dynamics of the infection, and much more needs to be done in whole animal models of chronic or repeated infection malaria infection, to test experimentally the contribution of T_reg_ to infection and disease processes during re-exposure. Finally, activation of T_reg_ cells urgently needs to be assessed in the context of longitudinal cohort studies in human populations to further test the association of T_reg_ activation parameters with respect to parasitological and pathophysiological outcome variables. Put together, these three avenues may be required to test the proposition that malaria actively engages in T_reg_ cell activation in order to promote the survival of the pathogen.
